# Evaluation of Vav3.1 as prognostic marker in endometrial cancer

**DOI:** 10.1007/s00432-018-2725-2

**Published:** 2018-08-06

**Authors:** Maximilian Boesch, Sieghart Sopper, Christian Marth, Heidi Fiegl, Annemarie Wiedemair, Julia Rössler, Jiri Hatina, Dominik Wolf, Daniel Reimer, Alain G. Zeimet

**Affiliations:** 1 Lungenzentrum, Kantonsspital St. Gallen, Rorschacherstrasse 95, 9007 St. Gallen, Switzerland; 2 Internal Medicine V, Medical University of Innsbruck (MUI), 6020 Innsbruck, Austria; 3 Tyrolean Cancer Research Institute (TKFI), 6020 Innsbruck, Austria; 4 Oncotyrol, Center for Personalized Cancer Medicine GmbH, 6020 Innsbruck, Austria; 5 Department of Gynecology and Obstetrics, Medical University of Innsbruck, Anichstrasse 35, 6020 Innsbruck, Austria; 6 Department of Biology and Biomedical Centre, Faculty of Medicine Pilsen, Charles University Prague, 30100 Pilsen, Czech Republic; 7 Medical Clinic III, Oncology, Hematology and Rheumatology, University Clinic Bonn (UKB), 53127 Bonn, Germany

**Keywords:** Vav3, Guanine nucleotide exchange factor, Cancer, Endometrial cancer, Transcript variant, Protein isoform

## Abstract

**Purpose:**

Vav3 is a guanine nucleotide exchange factor that regulates the activity of Rho/Rac family GTPases. In a study on ovarian cancer, we recently demonstrated pronounced prognostic and predictive value of Vav3.1, a specific truncation variant of the parental Vav3 gene. Here, we sought to investigate the role of Vav3.1 in the most prevalent gynecological tumor entity, endometrial cancer.

**Methods:**

Vav3.1 transcript levels were determined in a large cohort of endometrial cancer patients using variant-specific PCR (*n* = 239), and non-malignant endometrial tissue served as control (*n* = 26). Expression levels of Vav3.1 were stratified according to established clinicopathological characteristics and correlated to long-term patient survival (average follow-up of > 7.5 years). Type 1 and type 2 cancers were separately investigated.

**Results:**

While Vav3.1 was markedly overexpressed in endometrial cancer tissue, we could not detect associations with clinical parameters related to prognosis, such as FIGO stage and tumor grade. Kaplan–Meier estimators of different measures of survival failed to show prognostic significance of Vav3.1 in endometrial cancer. Lack of prognostic value was observed for both type 1 and type 2 cancers.

**Conclusions:**

Our study shows that Vav3.1 is not suited as a marker of cancer progression and/or treatment response in endometrial cancer. Feasibility and potential benefit of targeting Vav3.1 in endometrial cancer needs to be evaluated in future studies, proceeding from its clear, roughly ten-fold, induction in the malignant endometrium.

## Introduction

Endometrial cancer (EC) is the most prevalent gynecological malignancy and has traditionally been classified into two major subtypes that differ in histological appearance and clinical behavior (Hecht and Mutter 2006). The much more common type 1 cancers (accounting for roughly 80% of cases), which show endometrioid morphology, are associated with unopposed estrogen exposure, and frequently arise from premalignant lesions (Hecht and Mutter 2006; Setiawan et al. 2013). In contrast, type 2 cancers are of non-endometrioid histology (usually serous or clear cell), depend on hormone stimulation to a lesser extent, typically arise in the aged atrophic endometrium, and are often poorly differentiated (Hecht and Mutter 2006; Setiawan et al. 2013). Accordingly, type 2 cancers generally exhibit a worse clinical outcome (Setiawan et al. 2013). Despite this, recent evidence suggests that the etiologies of type 1 and type 2 cancers are more similar to each other than previously thought, with many risk factors shared including nulliparity, early menarche, and obesity (Setiawan et al. 2013).

Compared with other gynecological tumors, EC shows a reasonably good prognosis even at more advanced stages. Nevertheless, novel markers of progression and treatment response are desirable to refine patient stratification for further improvement of survival rates. Moreover, certain patient subgroups exhibit poor outcome despite low-risk classification according to *Fédération Internationale de Gynécologie et d’Obstétrique* (FIGO) staging, and filtering out these individuals is critical to consider additional therapeutic options early on hence preventing undertreatment of a whole patient subset (Zeimet et al. 2013).

Vav3 is a guanine nucleotide exchange factor (GEF) with specificity for Rho/Rac family GTPases (Movilla and Bustelo 1999), thus functioning as a regulator of cell motility, proliferation and differentiation (Hornstein et al. 2004). In addition, Vav3 is implicated in receptor-triggered angiogenesis (Hunter et al. 2006), and host deficiency in Vav2/Vav3 retards tumor growth based on impaired vascularization within the tumor bed (Brantley-Sieders et al. 2009). Vav3 has also been shown to mediate receptor tyrosine kinase signalling (Zeng et al. 2000) and to co-activate female (Lee et al. 2008) and male (Rao et al. 2012) hormone receptors. Importantly, Vav3 can induce cell transformation (Zeng et al. 2000) and targeted overexpression of Vav3 in prostatic epithelium induces tumor formation in mice (Liu et al. 2008). Consequently, Vav3 meets the key criterion of a bona fide oncogene. In the clinical setting, Vav3 is associated with cancer progression and recurrence (Lin et al. 2012) and furthermore mediates resistance to certain treatments, such as breast cancer endocrine therapy (Aguilar et al. 2014). Hence, Vav3 constitutes an established marker for cancer with prognostic and predictive significance, and accumulating evidence suggests a causal role in tumorigenesis that might be therapeutically exploitable.

Aside from Vav3, the mammalian Vav family comprises further two members, Vav1 and Vav2. Vav1 is specific for hematopoietic cells, whereas both Vav2 and Vav3 are more broadly expressed including expression in various epithelial and mesenchymal cell types (Bustelo 2000; Hornstein et al. 2004). More importantly, Vav3 can be subdivided into two main forms produced by alternative splicing, the full-length Vav3 alpha and the N-terminally truncated Vav3.1 (Boesch et al. 2018; Reimer et al. 2017; Trenkle et al. 2000). The latter form is devoid of several conserved domains including the region responsible for GEF activity, but has otherwise retained the potential for protein–protein interactions based on preserved SH2/SH3 motifs. The molecular properties of this isoform are virtually unknown, but dominant-negative effects on the full-length Vav3 alpha, or other modulating function within the Rho/Rac signalling circuit, are conceivable (Boesch et al. 2018).

In a study on ovarian cancer (OC), the most lethal gynecological tumor type (Cannistra 2004; Partridge and Barnes 1999), we recently established Vav3.1 as an important biomarker (Boesch et al. 2018; Reimer et al. 2017). High transcript levels of Vav3.1 were associated with poor overall survival (OS) and disease-free survival (DFS). Furthermore, abundant Vav3.1 message identified those patients that were refractory to platinum-based chemotherapy, the standard-of-care for OC patients. Of note, these analyses were inspired by an in-depth molecular profiling of the stem-like OC side population (SP) (Boesch et al. 2014) that showed upregulation of Vav3.1 especially in tumorigenic cells. Hence, the data suggested that CSC-specific/-enriched signatures bear prognostic and predictive significance in OC.

EC is stem cell-driven as well (Hubbard et al. 2009; Rutella et al. 2009), and evidence suggests that at least a sub-fraction of endometrial CSCs reside in the SP (Friel et al. 2008). However, EC is much less aggressive than OC and relapse rates are comparatively low (Kitchener et al. 2009; Notaro et al. 2016; Zeimet et al. 2013). This argues for significant differences in the underlying biology including (1) the degree of cellular heterogeneity and (2) the relative contribution of CSCs to disease progression. Hence, it remains unclear whether Vav3.1, as a factor potentially specific for CSCs, can serve as biomarker in EC. Here, we addressed this question in a monocentric retrospective study involving 239 patients.

## Materials and methods

### Patient samples

Tumor specimens were collected from EC patients undergoing primary surgery at the Department of Gynecology and Obstetrics at the Medical University of Innsbruck between the years 1989 and 2015 (*n* = 239). Endometrium samples from patients undergoing hysterectomy for reasons other than malignancy served as control (*n* = 26). Tissues were snap-frozen and pulverized directly after surgery and preserved at − 80 °C until RNA extraction. Patients have not been pre-selected or stratified according to clinicopathological risk factors, and there was no age-related cut-off.

### Clinicopathological characteristics

Patients were monitored within the regular outpatient follow-up program of the Department of Gynecology and Obstetrics at the Medical University of Innsbruck, with an average observation time of 7.57 years (range 0.08–25.75). Tumors were staged according to the FIGO classification system (Werner et al. 2012), and histological subtype and grading were determined based on WHO criteria. The majority of patients were FIGO stage I (roughly two-thirds) and most of them received radiation therapy after surgical debulking (> 85%). In contrast, only around 20% of patients received adjuvant chemotherapy, with the most commonly used regimens being platinum- and taxane-based drugs, or combinations thereof. As expected, the vast majority of patients showed endometrioid histology (type 1 cancers; >80%), while the remaining fraction exhibited either type 2 disease (serous/clear cell) or a mixed Müllerian phenotype. Patient characteristics are summarized in Table 1. OS was defined as the time from surgery to the last follow-up or until death from any cause. DFS was defined as the time from surgery to relapse or until death from any cause. Follow-up information was available for all patients.

**Table 1:** Patient characteristics (*n* = 239)

Characteristics	Median (range)
Age (years)	68.80 (36.52–92.92)
Overall survival (months)	69.00 (1.00–309.00)
Disease-free survival (months)	44.00 (0.00–280.00)

	*n*	%
FIGO stage^a^		
I	155	64.85
II	15	6.28
III	59	24.69
IV	9	3.77
Unknown	1	0.42
Histological subtype		
Endometrioid (type 1)	201	84.10
Serous/clear cell (type 2)	23	9.62
Mixed Müllerian	15	6.28
Histopathological grading		
1	43	17.99
2	122	51.05
3	74	30.96
Surgical resection/debulking		
Yes	239	100.00
No	0	0.00
Radiation therapy		
Yes	206	86.19
No	33	13.81
Chemotherapy		
Yes	49	20.50
No	190	79.50

^a^Fédération Internationale de Gynécologie et d’Obstetrique

### RNA extraction and generation of cDNA

Total RNA was extracted from pulverized tissue using a commercial kit (RNAgents^®^ Total RNA Isolation System; Promega, Fitchburg, WI), and samples were quality-controlled using gel analysis of ribosomal RNA (18S and 28S bands). Contaminating genomic DNA was eliminated through treatment with DNAse (Roche, Basel, Switzerland), and cDNA was generated using random hexamer priming. Reaction conditions and cocktail composition are specified in a previous publication of ours (Reimer et al. 2007).

### Primers and probes

The TaqMan^®^ Gene Expression Assay Hs00610104_m1 (Applied Biosystems, Foster City, CA) was used to determine Vav2 expression levels (NM_001134398.1). To analyse TBP (housekeeper) and the Vav3 variants Vav3 alpha (full-length; NM_006113.4) and Vav3.1 (5′-truncated; NM_001079874.1), we designed specific primers and probes using Primer Express^®^ software (Applied Biosystems) (Reimer et al. 2017). To avoid amplification of contaminating genomic DNA, primers were placed to span exons. Discrimination of Vav3 variants was accomplished positioning the forward primers between exons 18b and 19 (Vav3 alpha) and between exons 18a and 19 (Vav3.1). The reverse primer and probe were the same for both variants and were positioned within exon 20 and between exons 19 and 20, respectively. Primer and probe sequences for TBP, Vav3 alpha and Vav3.1 can be retrieved from (Reimer et al. 2017).

### Quantitative real-time PCR

Amplification of cDNA templates was performed on an ABI PRISM 7900HT Sequence Detection System run by SDS 2.3 software (both from Applied Biosystems). Reactions were run in a volume of 25 µl and contained 12.5 µl TaqMan^®^ Universal PCR Master Mix, 50 ng cDNA template, 900 nM of forward and reverse primer, respectively, and 250 nM of probe. The reaction conditions were a priming step of 50 °C for 2 min, a denaturing step of 95 °C for 10 min, and 45 cycles of 95 °C for 15 s and 65 °C for 1 min. All reactions were performed in triplicate with the mean value being used for subsequent calculation. Relative quantification of gene expression was done using the comparative C_T_ method and normalization to TBP. Amplification efficiencies were estimated based on external calibration with the ovarian cancer cell line HTB-77 (Reimer et al. 2017), and only experiments with an efficiency of > 95% were included in the study.

### Ethics statement

All patients gave written informed consent for use of their tissue in research. The study was approved by the local Institutional Ethical Review Board.

### Statistical analysis

Differences between two groups were analysed using the Wilcoxon–Mann–Whitney test (e.g., cancer vs. healthy) or the Wilcoxon signed-rank test (Vav3/Vav3.1 matched-pairs). Differences between three or more groups were analysed using the Kruskal–Wallis test with Dunn’s correction for multiple comparisons (e.g., FIGO stage and tumor grade). Results are presented as individual data points with the median value indicated. Potential associations between transcript levels and patient survival were evaluated using Spearman correlation statistics. For survival analyses taking into account censoring, the Kaplan–Meier estimator was used along with log-rank statistical testing. GraphPad Prism version 7 (GraphPad Software, Inc., La Jolla, CA) was used for statistical testing. Tests were two-sided and a *p* value < 0.05 was considered significant.

## Results

### Upregulation of Vav3.1 in endometrial cancer

**Fig. 1:**
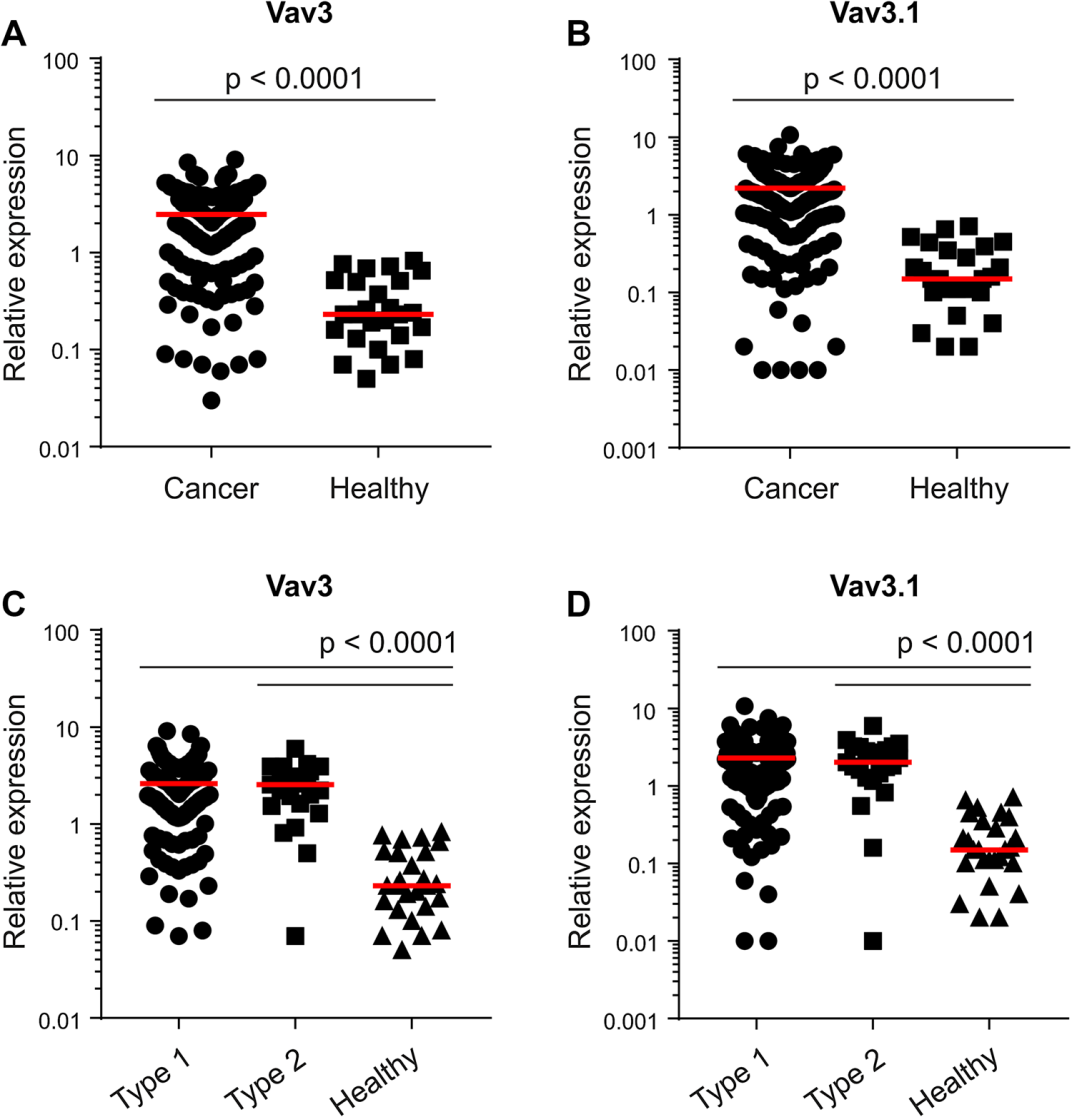
Overexpression of Vav3/Vav3.1 in endometrial cancer. Transcript levels of Vav3 (**a**) and Vav3.1 (**b**) were determined in endometrial cancer tissue and healthy control tissue using quantitative real-time PCR. Vav3 (**c**) and Vav3.1 (**d**) expression was also separately analysed in type 1 and type 2 cancers. Red lines indicate median values

To discriminate between 5′-truncated Vav3.1 and Vav3 (covering both full-length Vav3 alpha and Vav3.1), we applied variant-specific PCR (to our knowledge, no Vav3.1-selective antibody exists). In addition, we determined the transcript levels of Vav2, another member of the Vav family showing non-hematopoietic expression. As compared to healthy controls, both Vav3 (Fig. 1a) and Vav3.1 (Fig. 1b) were highly upregulated in EC with an increase in median expression of more than ten-fold, respectively (*p* < 0.0001). In contrast, we found that Vav2, which was measured in a sub-fraction of patients (*n* = 90), was only marginally upregulated in the cancer specimens (1.12-fold increase, *p* = 0.0387) (Suppl. Figure 1). Of note, overexpression of Vav3/Vav3.1 was equally pronounced in type 1 (endometrioid) and type 2 (serous/clear cell) cancers (Fig. 1c, d), indicating a common mechanism of Vav3 activation among principal EC sub-entities. Moreover, a paired analysis revealed great concordance between Vav3 and Vav3.1 expression in the individual patient, with Vav3 yielding only a slightly higher signal (median value 2.47 vs. 2.20, *p* < 0.0001) (Suppl. Figure 2). Together, these data provide evidence for significant upregulation of Vav3 and Vav3.1 in EC. The concordant expression pattern of Vav3 and Vav3.1 in individual patients suggests that most of the intratumoral Vav3 message originates from truncation variant Vav3.1, with only a minor contribution from full-length Vav3 alpha.

### Independence from clinicopathological characteristics

**Fig. 2:**
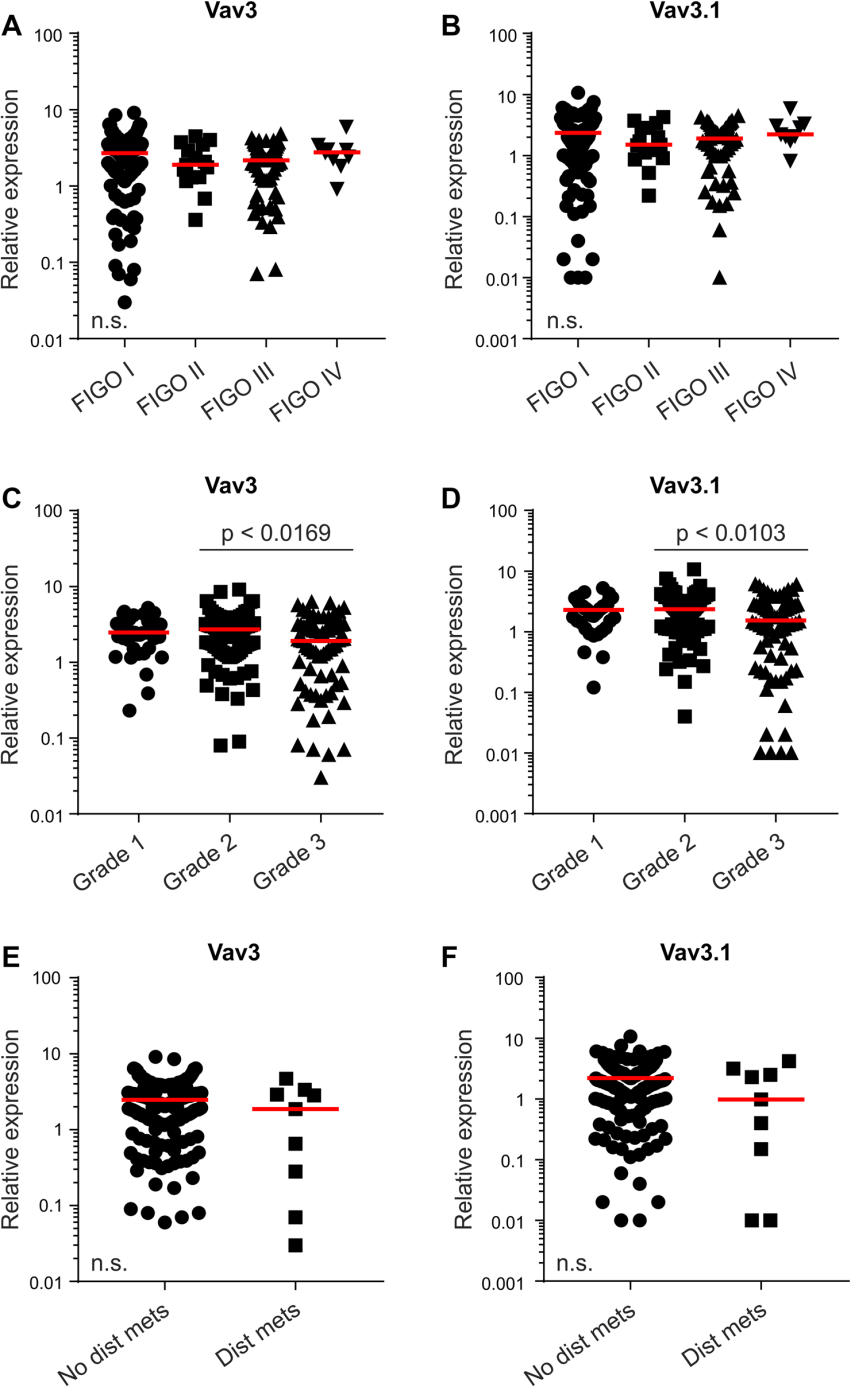
Vav3/Vav3.1 Expression is independent from clinicopathological characteristics. Patients were classified according to FIGO stage (**a, b**), tumor grade (**c, d**) or metastasis status (**e, f**), and Vav3 and Vav3.1 transcript levels were determined using quantitative real-time PCR. Red lines indicate median values. *Dist mets* distant metastases, *FIGO Fédération Internationale de Gynécologie et d’Obstétrique*. n.s. (*p* > 0.05)

Overall, we observed a high variation in Vav3/Vav3.1 transcript levels in our patient cohort (Table 1), which ranged from 0.01 to 10, thus spanning about three log decades (Fig. 1a–d and Suppl. Figure 2). We thus sought to test a possible association with established clinicopathological parameters, to see whether Vav3/Vav3.1 expression levels can discriminate between discrete patient subgroups. However, expression of Vav3/Vav3.1 did not change with FIGO stage (Fig. 2a, b), and there was no appreciable association with tumor grade (Fig. 2c, d). The same results were obtained in a separate analysis of type 1 and type 2 cancers (data not shown). Finally, Vav3/Vav3.1 levels also did not discriminate the subgroup of distantly metastasized patients (Fig. 2e, f), thus questioning a pronounced role in dissemination and systemic disease. Collectively, these data suggest that upregulation of Vav3/Vav3.1 expression in EC tissue occurs independently from various clinicopathological features that bear prognostic and/or predictive significance.

### Vav3.1 levels fail to predict survival of endometrial cancer patients

**Fig. 3:**
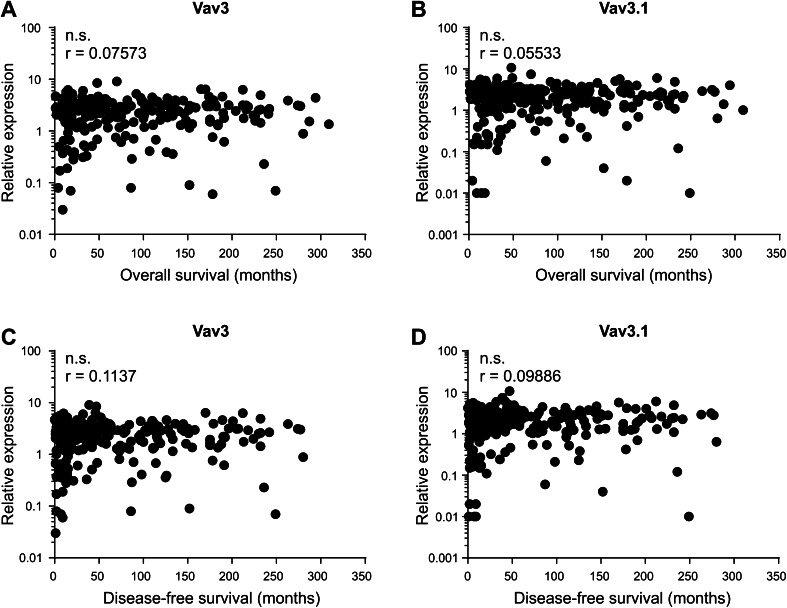
No correlation of Vav3/Vav3.1 expression with endometrial cancer survival. Vav3/Vav3.1 transcript levels were analysed for potential correlation with overall survival (**a, b**) and disease-free survival (**c, d**) using Spearman statistics. n.s. (*p* > 0.05)

**Fig. 4:**
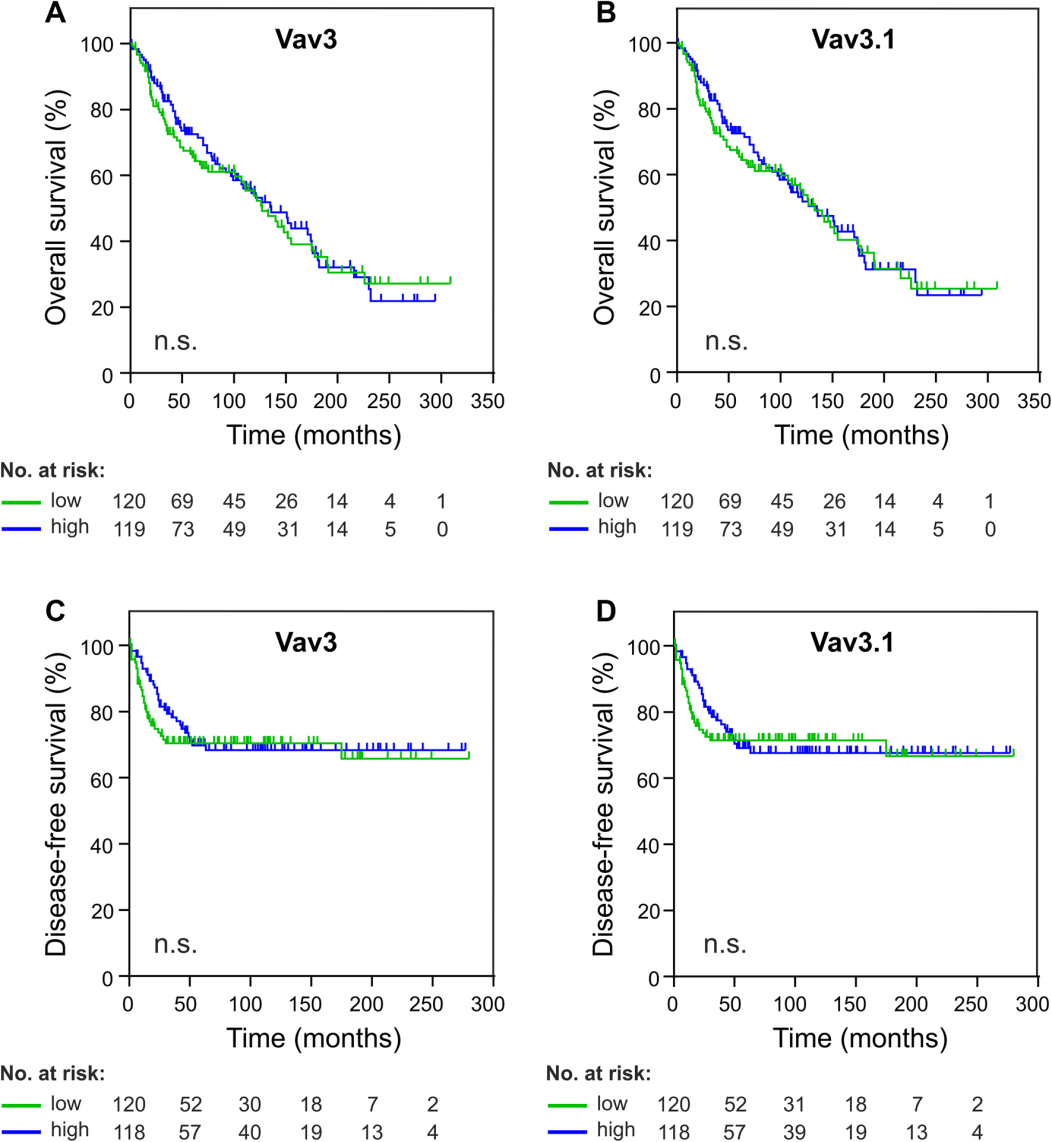
Vav3/Vav3.1 expression fails to predict endometrial cancer survival. Patients were dichotomized according to 50th percentile statistics and Kaplan–Meier survival analysis was performed for overall survival (**a, b**) and disease-free survival (**c, d**). The number of individuals at risk is indicated. n.s. (*p* > 0.05)

Lack of association with clinicopathological characteristics does not rule out independent prognostic relevance of a molecular marker. We, therefore, assessed whether Vav3/Vav3.1 transcript levels correlated with patient survival (OS and DFS). In an uncensored analysis, we could not discover significance of correlation between Vav3/Vav3.1 expression and patient survival, neither in pooled analyses (Fig. 3a–d) nor upon separation of type 1 and type 2 cancers (data not shown). Accordingly, Kaplan–Meier estimators taking into account censoring failed to demonstrate prognostic significance of Vav3/Vav3.1 expression after sectioning according to 50th percentile statistics (Fig. 4a–d). We obtained the same results for other percentiles as well (data not shown). These data consistently show that neither Vav3 nor Vav3.1 is significantly associated with patient survival, indicating that neither transcript is suitable as a prognostic tool to predict EC patient outcome.

## Discussion

EC is the most common gynecological cancer (Hecht and Mutter 2006) and exhibits a fairly good prognosis even at more advanced stages. Notwithstanding, low-risk classification based on FIGO I staging does not reliably identify patients with excellent prognosis who are indeed protected from recurrence (Zeimet et al. 2013). Thus, certain patient subgroups are at risk of undertreatment and novel prognostic markers are desirable to refine outcome prediction for a better initial stratification.

In a study on OC, we recently established prognostic significance of Vav3.1 in all major clinical subtypes (Boesch et al. 2018; Reimer et al. 2017). In addition, we found that high levels of Vav3.1 filtered out those patients that never responded to platinum-based treatment (i.e., genuine platinum refractoriness). Importantly, this retrospective study followed an exploratory analysis that linked Vav3.1 expression to phenotypic and functional CSC properties (overexpression in OC SP cells). Here, we tried to translate these findings to a gynecological tumor entity characterized by a much less aggressive clinical course.

The results from this large monocentric study demonstrate that both Vav3 and its transcript variant Vav3.1 are highly upregulated in EC. In addition, we discovered great concordance of Vav3 (covering both full-length Vav3 alpha and 5′-truncated Vav3.1) and Vav3.1 transcript levels in individual patients, suggesting that overexpression of Vav3 in tumor tissue is mainly due to Vav3.1, similarly as in OC (Reimer et al. 2017). Nevertheless, thorough investigations failed to demonstrate significant association with clinicopathological parameters, and the transcripts were also not able to predict EC patient survival. We, therefore, conclude that neither Vav3 nor Vav3.1 bears prognostic significance in EC. Other biomarkers, such as the recently discovered L1CAM (CD171) (Zeimet et al. 2013), therefore, remain unrivaled in prognosticating disease progression and recurrence in EC.

EC is associated with several recurrent mutations and other aberrations; however, the chronology of these events as well as the eventual genetic constellation underlies significant inter-patient variation (Hecht and Mutter 2006). Type 1 cancers often show defects in mismatch repair genes as well as specific mutations in *PTEN, K-ras* and *β-catenin* (Hecht and Mutter 2006; Kitchener et al. 2009). On the other hand, the much less common type 2 cancers frequently exhibit aneuploidy genotypes as well as mutations in the core tumor suppressor *TP53* (Hecht and Mutter 2006; Kitchener et al. 2009). Of note, the key role of *PTEN* in endometrial cancer suppression has been demonstrated and confirmed in pten^+/−^ mice, which develop endometrial hyperplasia and endometrial cancer with 100 and 20% penetrance, respectively (Stambolic et al. 2000). It is conceivable that in EC, Vav3.1 overexpression is regulated independently from these ‘driver’ mutations such that the level of expression does not correlate with clinicopathological features or survival. Alternatively, the detected Vav3.1 message might originate from non-malignant ‘bystander’ cells, whose presence may not be linked to tumor progression (e.g., particular populations of tumor-infiltrating immune cells, or stromal/endothelial cells).

Like most other organs of the adult, the endometrium represents a hierarchically organized tissue sustained and replenished by dedicated populations of stem cells (Verdi et al. 2014). In the endometrium, this stem cell pool appears to comprise cells of different lineages including epithelium, mesenchyme and endothelium, which is a quite special situation (Chan et al. 2004; Gargett and Masuda 2010). The endometrium also shows expression of embryonic stem cell antigens (Matthai et al. 2006) and interestingly, one of the lead candidate markers for the definition of endometrial stem cells is the SP phenotype (Friel et al. 2008; Masuda et al. 2015). There is evidence that the transformed endometrium harbors stem cell populations as well, which fuel malignant progression and escape the cytotoxic effects of chemotherapy; accordingly, clinical translation and therapeutic targeting of this cell pool is envisaged (Carvalho et al. 2015). Although several markers have been proposed for endometrial CSCs including CD133 (Rutella et al. 2009) and aldehyde dehydrogenase (van der Zee et al. 2015), it is striking to note that one of the most recurrently mentioned markers, again, is the SP phenotype (Friel et al. 2008; Gotte et al. 2011; Kusunoki et al. 2013).

In OC, we found that Vav3.1 enriched in CSCs is clinically relevant in predicting survival and treatment response (Boesch et al. 2018; Reimer et al. 2017). It might be that Vav3.1 is less specific for CSCs in EC and that accordingly, more differentiated tumor cells account for most of the detected signal. Alternatively, the relative abundance of CSCs might be lower in EC such that fewer cells contribute Vav3.1 message and CSC specificity of the signature is lost. Indeed, we found that the median expression of Vav3.1 was higher in OC (4.58) (Reimer et al. 2017) than in EC (2.2). In support of this hypothesis, OC is a particularly aggressive tumor type showing high rates of recurrence and frequent acquisition of drug resistance (Zeimet et al. 2012), both of which are associated with CSCs and tumoral stemness (Boesch et al. 2014, 2015, 2016a, b). In contrast, the relatively slow progression kinetics and favorable outcome of EC (Kitchener et al. 2009; Notaro et al. 2016; Zeimet et al. 2013) portend that this tumor entity may be less driven by CSCs. Assuming CSC specificity of the Vav3.1 transcript, this could at least partially explain their lack of association with patient survival and clinicopathological features in EC.

The fact that Vav3.1 cannot be harnessed for prognostic inferences in EC does not rule out the possibility that its targeting can confer therapeutic benefit. We thus suggest that in the effort of developing Vav3.1-targeting drugs, EC should not be forgotten as a potential indication, even though other tumor types will definitely be at the forefront of these investigations. In the meantime, it should be established on the level of genetic specificity whether Vav3.1 expression affects fundamental stem cell properties in EC, such as clonogenicity, tumorigenicity, and chemo-sensitivity.

Collectively, we here show that Vav3.1 does not serve as biomarker for EC progression, despite significant overexpression in tumor tissue. This might be a direct consequence of the rather lowly aggressive clinical behaviour of this tumor type which is indicative of an exhausted tumoral stemness potential or generally a small contribution from CSCs. Further research is required to see whether Vav3.1 targeting bears therapeutic potential in EC.

## Abbreviations

CSC: Cancer stem cell
DFS: Disease-free survival
EC: Endometrial cancer
FIGO: Fédération Internationale de Gynécologie et d’Obstétrique
GEF: Guanine nucleotide exchange factor
OC: Ovarian cancer
OS: Overall survival
SP: Side population
